# Correlation between antifungal consumption and the distribution of *Candida* species in different hospital departments of a Lebanese medical Centre

**DOI:** 10.1186/s12879-018-3512-z

**Published:** 2018-11-20

**Authors:** Lyn Awad, Hani Tamim, Dania Abdallah, Mohammad Salameh, Anas Mugharbil, Tamima Jisr, Kamal Zahran, Nabila Droubi, Ahmad Ibrahim, Rima Moghnieh

**Affiliations:** 1 Infectious Diseases and Antimicrobial Stewardship Clinical Pharmacist, Makassed General Hospital, Beirut, Lebanon; 2 Department of Internal Medicine, American University of Beirut, Beirut, Lebanon; 3 Pharmacy Department, Makassed General Hospital, Beirut, Lebanon; 4 Department of Internal Medicine, Makassed General Hospital, Beirut, Lebanon; 5 Division of Hematology/Oncology, Department of Internal Medicine, Makassed General Hospital, Beirut, Lebanon; 6 Department of Laboratory Medicine, Makassed General Hospital, Beirut, Lebanon; 7 Middle East Institute of Health, Bsalim, Beirut, Lebanon; 8 Pharmacy Department, Makassed General Hospital, Beirut, Lebanon; 9 Head of Antimicrobial Stewardship Program, Makassed General Hospital, Beirut, Lebanon

**Keywords:** Amphotericin B, Antifungal, Azoles, *Candida albicans*, *Candida glabrata*, *Candida famata*, Consumption, Correlation, Critical care, Echinocandins, Non-albicans *Candida*, Obstetrics, Oncology

## Abstract

**Background:**

In recent years, there has been a significant increase in the incidence of fungal infections attributed to *Candida species* worldwide, with a major shift toward non-*albicans Candida* (NAC). In this study, we have described the distribution of *Candida* species among different hospital departments and calculated the antifungal consumption in our facility. We also correlated the consumption of certain antifungals and the prevalence of specific *Candida species.*

**Methods:**

This was a retrospective review of all the *Candida* isolates recovered from the computerised microbiology laboratory database of Makassed General Hospital, a tertiary care centre in Beirut, Lebanon, between January 2010 and December 2015. Data on antifungal consumption between January 2008 and December 2015 were extracted from the hospital pharmacy electronic database. We used Spearman’s coefficient to find a correlation between *Candida* species distribution and antifungal consumption.

**Results:**

Between 2008 and 2015, we observed that the highest antifungal consumption was in the haematology/oncology department (days of therapy/1000 patient days = 348.12 ± 85.41), and the lowest was in the obstetrics/gynaecology department (1.36 ± 0.47). In general, the difference in antifungal consumption among various departments was statistically significant (*P* < 0.0001). Overall, azoles were the most common first-line antifungals in our hospital. Echinocandins and amphotericin B were mostly prescribed in the haematology/oncology department. As for *Candida* species distribution, a total of 1377 non-duplicate isolates were identified between 2010 and 2015. A non-homologous distribution of *albicans* vs. non-*albicans* was noted among the different departments (*P* = 0.02). The most commonly isolated NAC was *Candida glabrata,* representing 14% of total *Candida species* and 59% of NAC. *Candida famata* (9% of NAC), *Candida parapsilosis* (3.6% of NAC) and *Candida krusei* (3% of NAC) were recovered unequally from the different departments. The total antifungal consumption correlated positively with the emergence of NAC. The use of azoles correlated positively with *Candida glabrata*, while amphotericin B formulations correlated negatively with it. None of these correlations reached statistical significance.

**Conclusion:**

Different *Candida* species were unequally distributed among different hospital departments, and this correlated with consumption of antifungals in respective departments, highlighting the need for antifungal stewardship.

## Background

In recent years, the world has witnessed a significant increase in the incidence of fungal infections due to *Candida* species [1], with *Candida albicans* (CA) being the most common causative organism [2]. However, recent studies have documented a change in this aetiology shifting toward non-*albicans Candida* (NAC) [3]. This shift has been linked to the selective pressure caused by the extensive use of broad spectrum antibiotics and antifungals [4]. For many years, azoles have been used as prophylactic agents against fungal infections in immunocompromised patients, as empiric/preemptive treatment of fungal disease in cancer or critically ill patients, in addition to their use as targeted therapy of *Candida* infections [1, 5]. However, polyenes consumption, especially lipid formulations with amphotericin B, has been used increasingly in the immunocompromised population [6], along with echinocandins, which were introduced into the market in 2002 [7].

The geographic distribution of *Candida* species may reflect the antimicrobial prescription habits in each healthcare facility [8, 9]. Multiple studies have looked at the relative distribution of *Candida* species with time [3, 8, 10]; however, few have studied its geographic distribution (i.e., in specific hospital departments along with corresponding antifungal expenditure) [8, 11]. Two studies demonstrated an increase in the incidence of *Candida parapsilosis* associated with the use of caspofungin [12, 13].

In this study, our primary aim was to calculate the consumption of each class of antifungals, and to describe the relative distribution of different *Candida* species in different hospital departments. The secondary aim was to find a correlation between the expenditure of certain antifungals and the prevalence of specific *Candida* species.

## Methods

### Setting and study design

This was a retrospective review of all *Candida* isolates retrieved from the computerised microbiology laboratory database at Makassed General Hospital (MGH), between January 2010 and December 2015. The MGH Institutional Review Board Committee granted this study approval. No informed consent was required due to it retrospective nature. The reporting of this study conforms to the STROBE statement [14]. MGH is a 186-bed university hospital in Beirut, Lebanon. The monthly occupancy rate ranges between 70 and 80%, with 17 beds in critical care, 71 beds in internal medicine (IM), 17 beds in haematology/oncology and bone marrow transplantation, 21 beds in surgery, 13 beds in obstetrics/gynaecology (OBGYN) and 47 beds in the paediatric departments. All *Candida* species reported in the database were selected, and non-duplicate isolates recovered during the study period were included in this study. All specimens from different culture sites, such as abscess, bronchoalveolar lavage, blood, catheter, ear, eye, fluid, sputum, deep-tracheal aspirate, throat, urine, vagina and wound were included, except for stool specimens.

### Identification and speciation of Candida isolates

The identification and speciation of *Candida* isolates were performed according to the microscopic and macroscopic growth morphology and germ tube test. Isolates producing germ tubes within 3 h of incubation were further differentiated. Speciation of the *Candida* isolates was performed using the API 20 C AUX system (bio Merieux, France) [15]. Results were interpreted after 48 to 72 h of incubation at 29 °C ± 2 °C. Antifungal susceptibility testing is not available in our institution.

### Protocol for antifungal use in our hospital

Broad-spectrum antifungals were prescribed on hospital setting for the management of invasive fungal infections according to institutional guidelines on antimicrobial use, which were based on international guidelines [16, 17]. Patient colonisation with *Candida spp.* such as in urine, sputum, skin or others was not usually treated with antifungals. Patients with evidence of candidemia or other invasive *Candida* infections were treated with antifungals [16]. Neutropenic patients with cancer were given prophylactic, empiric, pre-emptive or targeted antifungal therapy as per the clinical need [17]. In ICU, newly diagnosed septic patients without an evident focus of infection were evaluated by an Infectious Disease physician for the possibility of adding on systemic antifungals to their treatment regimens in the following cases: having already received broad-spectrum antibacterial therapy active against our nosocomial flora and having been colonized with *Candida species*. Systemic infections due to *C. albicans* and *C. tropicalis* were managed using fluconazole, while other NAC infections except for those caused by *C. krusei* were treated using echinocandins [16, 18]. *C. krusei*-related infections were given lipid formulation amphotericin B [16, 18].

### Definitions

#### Non-duplicate isolates

When multiple isolates were obtained from the same patient, all species were included in the study, but only the first isolate of a given species was considered in the analysis [8].

#### Days of therapy (DOT)

The number of days that a patient was on an antimicrobial regardless of the dose [19].

#### Defined daily dose (DDD)

Corresponds to the assumed average daily dose of an antimicrobial for its main indication in adults based on the World Health Organisation Anatomical Therapeutic Chemical (WHO/ATC) classification system for each antifungal [20].

#### Patient days (PD)

Calculated by counting the number of patients present in any given location (e.g., hospital or ward) at a single time during a 24-h period [21].

### Antifungal consumption

Data on antifungal consumption between January 2008 and December 2015 were extracted from the electronic database of the hospital pharmacy. Antifungals were categorised according to their pharmacological class: azoles (fluconazole and voriconazole), echinocandins (caspofungin, anidulafungin, micafungin) and polyenes (conventional amphotericin B and lipid formulations amphotericin B). We used two types of metrics to measure antifungal expenditure: DDD/1000 PD, and DOT/1000 PD [20, 22].

In paediatrics, the use of DOT is preferred because the antimicrobial doses are adjusted according to body weight, and there is no universal DDD [22]. In order to compare the antifungal consumption in the different hospital departments, including the paediatric department DOT/1000 PD had to be used. In the critical care and haematology/oncology departments, we used DDD/1000 PD to compare and benchmark with other studies in the published literature.

The DDD was 200 mg for fluconazole, 400 mg for voriconazole, 100 mg for anidulafungin, 50 mg for caspofungin and 100 mg for micafungin [20]. 19 There is no standardised DDD for the lipid formulations of polyenes [22, 23]. There is only a DDD for the amphotericin B deoxycholate (Fungizone®), which is 35 mg. Thus, for lipid-based formulations, we defined the DDD based on the regular daily dose used in our facility, which is 300 mg for both the liposomal and the lipid complex formulations.

### Statistical analysis

The Statistical Package for Social Sciences (SPSS, version 21) program was used for data entry, management and analysis. Categorical variables are presented as number and percent, whereas continuous variables are presented as mean and standard deviation. Bivariate analysis was carried out using the chi-squared test for comparing categorical variables, whereas continuous ones were compared using the Student’s t-test. The relationship between antifungal usage and the distribution of NAC was determined using the Spearman’s coefficient for non-parametric correlation. A *P* < 0.05 was considered significant.

## Results

### Antifungal consumption

The rate of antifungal consumption in DOT/1000 PD and DDD/1000 PD are shown in Tables 1 and 2, respectively. All rates were reported as mean ± standard deviation. From 2008 to 2015, the mean total antifungal consumption, in terms of DOT/1000 PD was 180.69 ± 135.5.

**Table 1:** Antifungal consumption in terms of DOT/1000 PD (mean ± SD) among different hospital departments between 2008 and 2015

Antifungal Class	Hospital Department						
	Critical care	*Paediatric*	Hem/Onc	OBGYN	Surgery	IM	*P*-value
Azoles	48.41 ± 14.58	23.29 ± 9.24	214.65 ± 47.67	0.94 ± 1.10	24.31 ± 10.24	46.44 ± 9.50	< 0.0001
Echinocandins	21.13 ± 27.26	1.35 ± 1.55	67.96 ± 19.04	0.42 ± 1.19	7.20 ± 11.25	7.69 ± 5.49	< 0.0001
Amphotericin B	4.32 ± 3.62	4.06 ± 3.36	65.50 ± 18.98	0	2.06 ± 3.62	2.34 ± 1.49	< 0.0001
Total	73.85 ± 22.25	28.70 ± 11.96	348.12 ± 85.41	1.36 ± 0.47	33.56 ± 11.65	56.48 ± 24.06	< 0.0001

KEY: *Hem/Onc* = Haematology/oncology, *IM* = Internal medicine, *OBGYN* = Obstetrics/gynaecology

**Table 2:** Antifungal consumption in terms of DDD/1000 PD (mean ± SD) among different hospital departments between 2008 and 2015

Antifungal Class	Hospital Department				
	Critical care	Hem/Onc	OBGYN	Surgery	IM
Azoles	33.79 (±10.98)	146.51 (±41.28)	0.56 (±1.03)	22.76 (±9.97)	39.73 (±7.25)
Echinocandins	11.36 (±8.33)	57.96 (±25.48)	0	4.38 (±8.27)	7.26 (±6.92)
Amphotericin B	4.29 (±4.36)	79.83 (±30.56)	0	0.50 (±0.98)	1.93 (±1.60)

KEY**:** *Hem/Onc* = Haematology/oncology, *IM* = Internal medicine, *OBGYN* = Obstetrics/gynaecology

Results in the two metric methods (DOT/1000 PD and DDD/1000 PD) revealed almost parallel patterns, with the exception of the azoles because the used daily doses of azoles in the hospital were much lower than the DDD, and were not consistent among different indications.

In general, the rate of antifungal consumption during the study period was not analogous among the different departments (*P* < 0.0001) (Table 1). In terms of DOT/1000 PD, total antifungal consumption was highest in the haematology/oncology department (348.12 ± 85.41), followed by critical care (73.85 ± 22.25), and was lowest in the OBGYN department (1.36 ± 0.47) (Table 1). The difference between the mean antifungal consumption in any two departments was statistically significant when compared to one another, with the exception of the consumption in the surgery department (33.56 ± 11.65) when compared to that in the paediatric department (28.70 ± 11.96), and the critical care department (73.85 ± 22.25) when compared to that in the IM department (56.48 ± 24.06), where the difference was non-significant (*P* = 0.565 and 0.23, respectively). Relative azole consumption mirrored total antifungal consumption, where the difference was statistically significant among the departments (*P* < 0.0001). Likewise, the highest consumption was seen in the haematology/oncology department (214.65 ± 47.67), and the lowest was seen in the OBGYN department (0.94 ± 1.10) (Table 1). Echinocandins were mostly used in oncology (67.96 ± 19.04), followed by a significantly lower consumption in critical care (21.13 ± 27.26, *P* = 0.01), and almost a null consumption in the paediatric and OBGYN departments (1.35 ± 1.55 and 0.42 ± 1.19 respectively, *P* = 0.2) (Table 1). Finally, conventional or lipid formulations of amphotericin B were mostly used in the haematology/oncology department (65.50 ± 18.98), followed by a similar distribution in critical care and paediatric departments (4.32 ± 3.62, and 4.06 ± 3.36, respectively, *P* = 0.885), then to a lesser extent in the IM and surgery departments (mean 2.34 ± 1.49, and 2.06 ± 3.62, respectively, *P* = 0.840). It was never used in the OBGYN department (Table 1). When antifungal consumption was measured in DDD/1000 PD, a similar trend was observed, excluding the paediatric department (Table 2).

### Candida isolates distribution

**Fig. 1:**
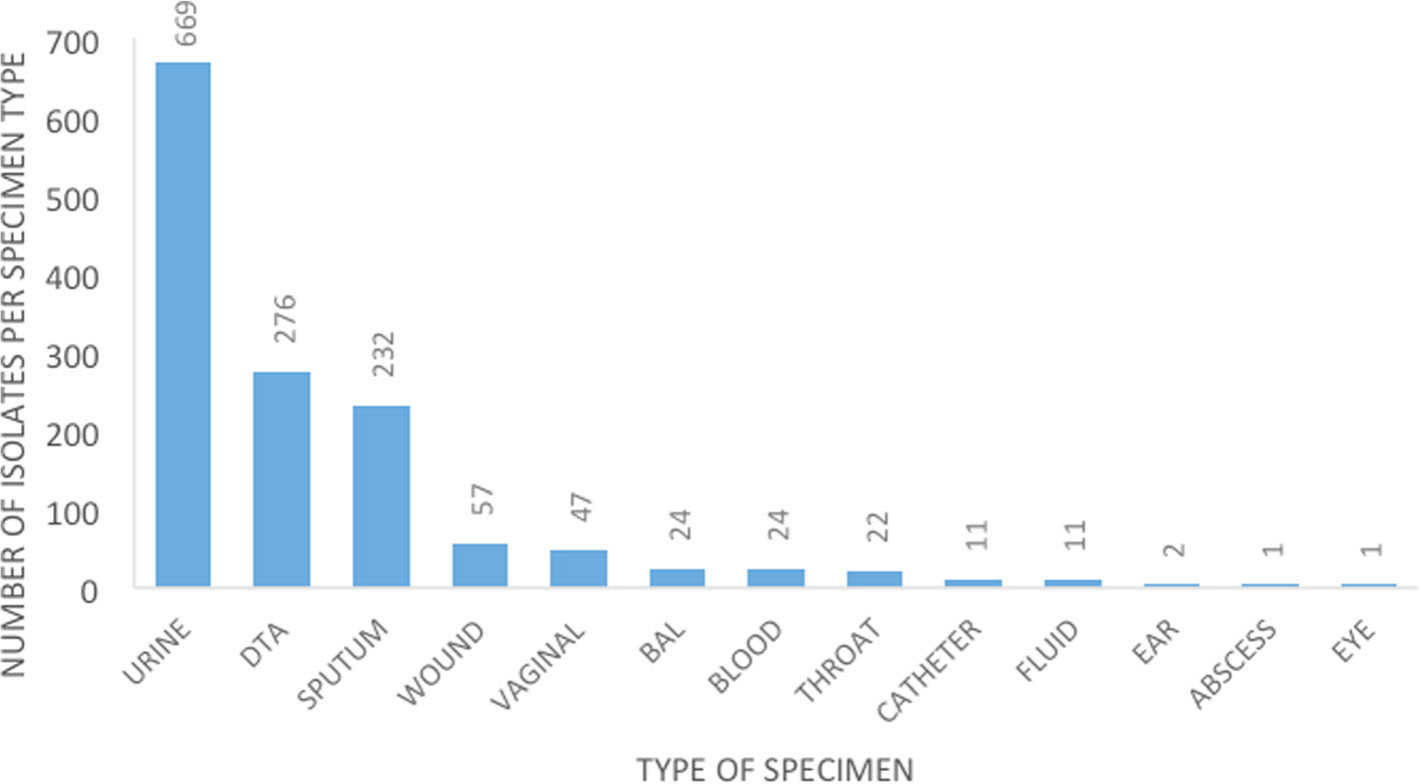
Distribution of different specimens types growing *Candida* species. KEY: BAL = Bronchoalveolar lavage, DTA = Deep tracheal aspirate

Between 2010 and 2015, a total of 1377 non-duplicate *Candida* isolates were identified, including colonizers and pathogens. The majority of these isolates were recovered from urine (48%), followed by the respiratory tract (20% from deep-tracheal aspirate and 17% from sputum), and only 2% were from blood (Fig. 1).

**Fig. 2:**
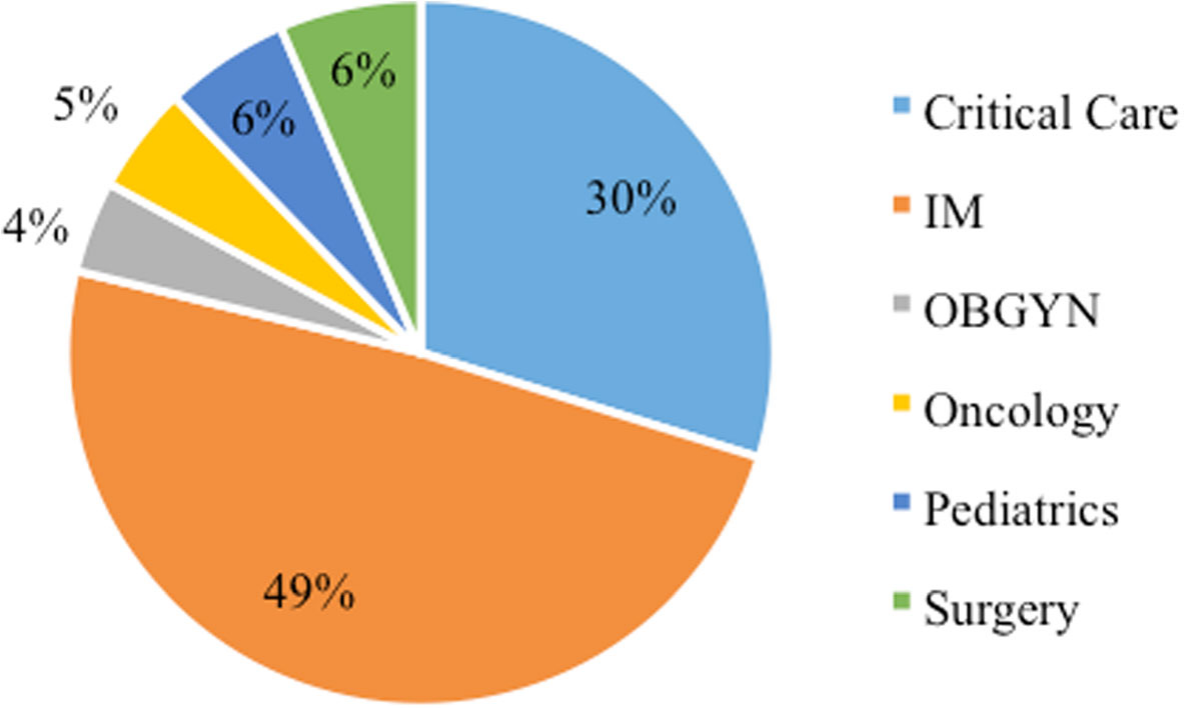
Distribution of *Candida* isolates in the different hospital departments. KEY: IM = Internal medicine, OBGYN = Obstetrics/gynaecology

The highest number of isolates was collected from the IM department (49%), followed by the critical care department (30%), the surgery (6%), paediatric (6%), haematology/oncology (5%) and OBGYN (4%) departments (Fig. 2).

### General distribution of Candida species with a focus on NAC in different departments

In all departments and NAC isolation was statistically significant among hospital departments (*P* = 0.02) (Table 3).

**Table 3:** Distribution of *Candida albicans* versus non-albicans *Candida* among hospital departments between 2010 and 2015 and comparison between them

Department	*Candida albicans* (*N* = 1044 isolates)	Non-albicans *Candida* (*N* = 333 isolates)	P-value
Critical care	313 (76.3%)	97 (23.7%)	0.02
IM	498 (73.9%)	176 (26.1%)	
OBGYN	53 (91.4%)	5 (8.6%)	
Hem/Onc	50 (74.6%)	17 (25.4%)	
Paediatric	67 (84.8%)	12 (15.2%)	
Surgery	63 (70.8%)	26 (29.2%)	

KEY: *Hem/Onc* = Haematology/oncology, *IM* = Internal medicine, *OBGYN* = Obstetrics/gynaecology

**Fig. 3:**
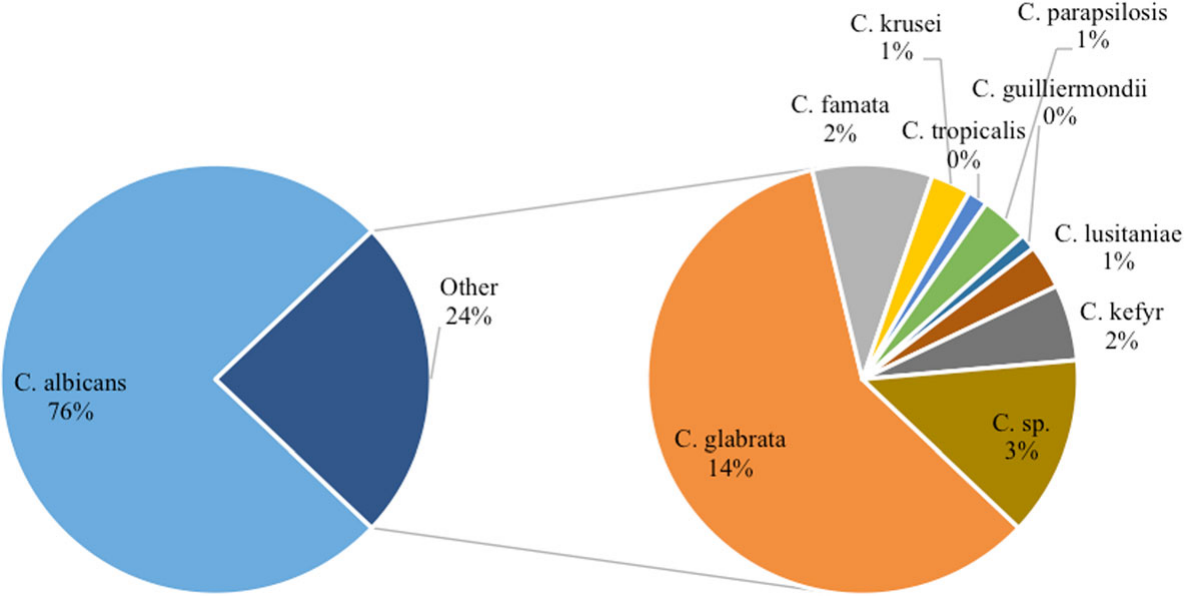
Total distribution of the different *Candida* species in the hospital. KEY: C. sp. = Non-speciated *Candida*. N.B. Percentages are calculated from total *Candida* isolates recovered

In all departments, CA was the most commonly isolated species, representing 76% of total isolates (Fig. 3) and ranging from 91.4% in the OBGYN department to 70.8% in the surgery department (Table 4).

**Table 4:** Distribution of the different *Candida* species (number of isolates, %) among different wards between 2010 and 2015

Ward	Total	Total NAC	*C. albicans* (*n* = 1044)	*C. glabrata* (*n* = 197)	*Non-speciated C.* (*n* = 45)	*C. famata* (*n* = 30)	C. Kefyr (*n* = 19)	*C. parapsilosis* (*n* = 12)	*C. lusitaniae* (*n* = 11)	*C. krusei* (*n* = 10)	*C. tropicalis* (*n* = 5)	*C. guilliermondii* (*n* = 4)	*P*
Critical care	410	97	313 (76%)	60 (62%)	12 (12%)	12 (12%)	5 (5%)	3 (3%)	1 (1%)	3 (3%)	0	1 (1%)	0.005
IM	674	176	498 (74%)	106 (60%)	25 (14%)	11 (6%)	11 (6%)	5 (3%)	10 (6%)	4 (2%)	4 (2%)	0	
OBGYN	58	5	53(91%)	5 (100%)	0	0	0	0	0	0	0	0	
Hem/Onc	67	17	50 (75%)	8 (47%)	1 (6%)	4 (23%)	0	0	0	1 (6%)	1 (6%)	2 (12%)	
Pediatrics	79	12	67 (85%)	4 (33%)	3 (25%)	1 (8%)	1 (8%)	3 (25%)	0	0	0	0	
Surgery	89	26	63 (71%)	14 (54%)	4 (15%)	2 (8%)	2 (8%)	1 (4%)	0	2 (8%)	0	1 (4%)	

KEY: *Hem/Onc* = Haematology/oncology, *IM* = Internal medicine, *NAC* = non-albicans *Candida*, *OBGYN* = Obstetrics/gynaecology

N.B**.** Percentages for *Candida albicans* are calculated from total isolates in each ward. Percentages for each of the non-albicans species are calculated from total NAC of each ward

In terms of NAC, the OBGYN department had the lowest significant rate of NAC compared to the critical care, haematology/oncology and surgery departments (*P* = 0.01, 0.02, and 0.003 respectively).

The rate of NAC in the paediatric department was the second lowest compared to other hospital units (Table 4).

### Distribution of different species of NAC

**Fig. 4:**
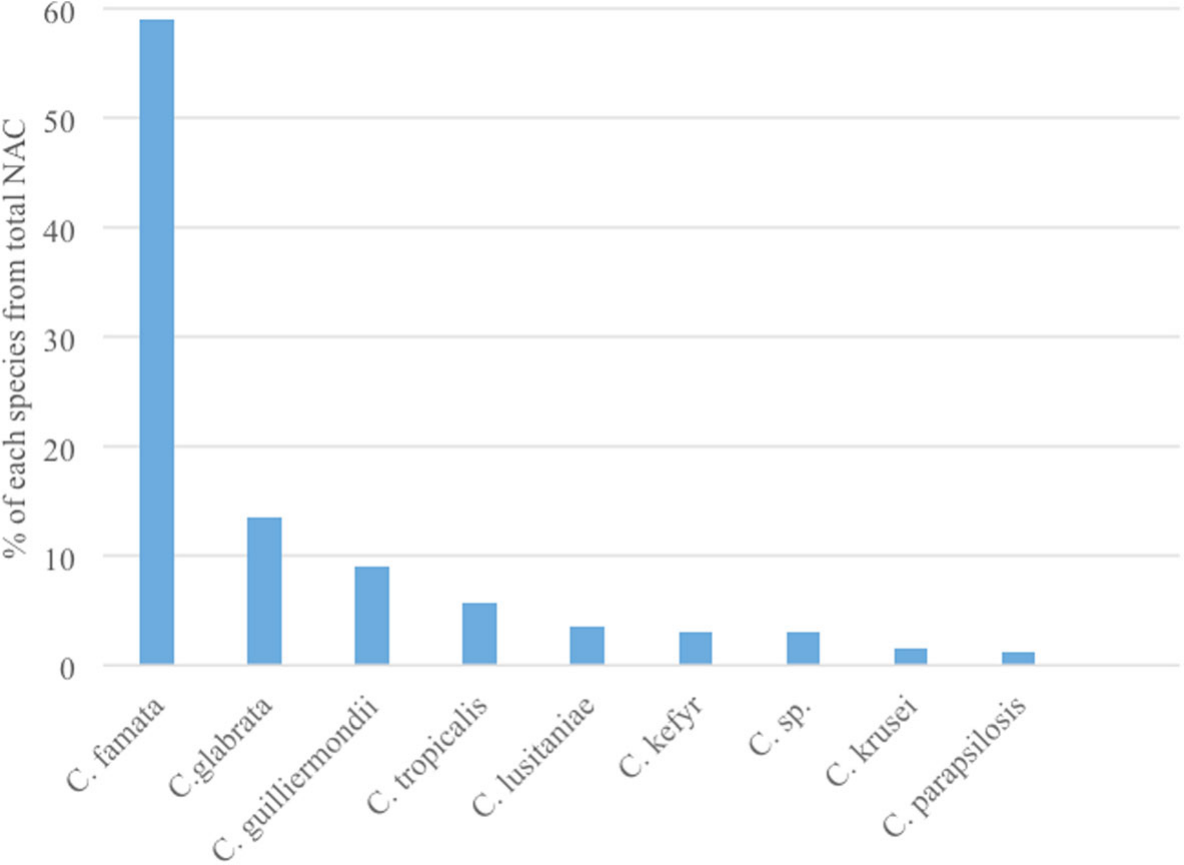
Percentages of the different species of NAC from total NAC. KEY: C. sp. = Non-speciated *Candida*

The most commonly isolated NAC in our facility was *Candida glabrata* (197 isolates), accounting for 14% of total isolates and 59% of total NAC. Non-speciated *Candida* followed, accounting for 3% of total isolates and 13.5% of total NAC. *Candida famata* came in third place, representing 2% of total isolates and 9% of total NAC. *Candida krusei* and *Candida tropicalis* represented 1 and 0.5% of the total isolates, respectively (Figs. 3 and 4).

### Distribution of Candida glabrata in different departments

The proportion of *Candida glabrata* among NAC differed among the units. In OBGYN, *Candida glabrata* has been the only isolated NAC (100% of total NAC). The lowest rate of *Candida glabrata* among NAC was seen in the paediatric department (33% of NAC), and the haematology/oncology departments (47% of NAC) (Table 5) (*P* = 0.03).

**Table 5:** Correlation of antifungal consumption (based on mean DOT/1000 PD) and non-albicans *Candida* isolation

		Non-albicans *Candida*	*Candida glabrata*
Total antifungals	Spearman’s coefficient	0.38	0.12
	p-value	0.46	0.83
Azoles	Spearman’s coefficient	0.40	0.13
	p-value	0.43	0.80
Echinocandins	Spearman’s coefficient	0.39	0.18
	p-value	0.43	0.73
Amphotericin B	Spearman’s coefficient	0.27	−0.003
	p-value	0.59	0.99

N.B. A positive value (Spearman’s coefficient) represents a positive correlation and a negative value (Spearman’s coefficient) represents a negative correlation

### Distribution of NAC other than Candida glabrata

The proportion of NAC other than *Candida glabrata* among different departments is presented in Table 3. There was less variability in the type of NAC in the OBGYN, haematology/oncology and paediatric departments in comparison with the IM, critical care and surgery departments.

The non-speciated *Candida* were mostly observed in the paediatric department (25% of NAC), followed by the surgery (15% of NAC), IM (14% of NAC), critical care (12.4% of NAC) and oncology departments (6% of NAC). They were absent in the OBGYN department.

Second to *Candida glabrata* among the speciated *Candida*, *Candida famata* (9% of NAC) was the most common in all departments, with the exceptions of the paediatric and OBGYN departments.

*Candida krusei* (3% of NAC) was mostly recovered from the surgery and haematology/oncology departments, while it was absent from the OBGYN and paediatric departments.

*Candida parapsilosis* (3.6% of NAC) had the highest percentage among the speciated *Candida* isolates second to *Candida glabrata* in the paediatric department (25%).

### Correlation between antifungal consumption and the isolation of specific Candida species

Using Spearman’s coefficient, we observed that none of the correlations reached a statistical significance due to the limited number of hospital departments involved (*N* = 5); however, these results indicated some trends and showed clinical significance (Table 5).

The use of antifungals in general correlated positively with NAC [Spearman’s Coefficient (SC) = 0.38]. The use of amphotericin B showed a similar yet weaker positive correlation for the emergence of NAC in comparison with azoles and echinocandins (SC = 0.27 vs. 0.40 and 0.39, respectively). The effect of azoles and echinocandins on recovery of NAC was almost the same.

Regarding *Candida glabrata* alone, the use of azoles correlated positively with its emergence (SC = 0.13), unlike the use of amphotericin B, which correlated negatively (SC = − 0.003).

We noticed that in wards where amphotericin B had been used (haematology/oncology and paediatrics, 19 and 14% from total antifungal consumption in each ward, respectively), there was less non-speciated *Candida* isolated from clinical specimens (Tables 1 and 5). We equally noticed less variability in the types of NAC recovered.

## Discussion

The effect of antimicrobial use on the changing microbial ecology of inpatients in a specific healthcare facility is sure but slow, and the tangible consequences usually lag behind in time [24]. Accordingly, we reviewed antifungal consumption in different hospital departments from 2008 to 2015, and we studied the distribution of variable *Candida* species in the same departments during 2010 and 2015. Then, we attempted to find a correlation between the use of specific antifungals and the prevalence of specific *Candida* species.

### Antifungal consumption in different departments

The discrepancies found between DOT and DDD per 1000 PD were due to the actual dosing of antifungals, especially the oral dosage form of fluconazole. The actual fluconazole doses that were used during the study period were below the DDD (200 mg actual dosing vs. 400 mg recommended dosing) for indications, including oral thrush and vaginitis.

In Lebanon, like in the Middle East and North Africa region, there are limited data about antifungal consumption. In our study, azoles were the most commonly used antifungals. Similarly, Al Othman et al. studied the burden and treatment patterns of invasive fungal infections in Lebanon and in the Kingdom of Saudi Arabia, in which they found that fluconazole was the most commonly prescribed antifungal as a first-line therapy (69%) [25]. The most common second-line antifungals were voriconazole (35%)/caspofungin (30%), followed by amphotericin B formulations in general [25]. In Europe, a multicentre French survey in 2012 involving 239 healthcare facilities similarly revealed that fluconazole was the most frequently used antifungal agent in haematology units and intensive care units (ICUs) [26]. Antifungal expenditure recorded its highest levels in participating cancer centres, followed by university hospitals [26]. In our hospital, the haematology/oncology department showed the highest antifungal consumption.

The comparison of our antimicrobial expenditure with other studies is hindered by the fact that the metrics are not standardised across hospitals and countries. Some use the recommended daily dose (RDD)/100 PD, such as Germany [27]. Others use DDD/1000 PD as in the formerly stated French study [26]. Some use DDD/1000 inhabitants, such as the European Centre for Disease Prevention and Control [28]. In paediatrics, because the calculations become even more complicated due to weight-based dosing, we used DOT/1000 PD [22].

### CA as the most commonly isolated species

CA was the most commonly isolated species (76% of total isolates) in our setting. So far in Lebanon, only two studies have described the distribution and epidemiology of variable *Candida* species in different medical institutions [10, 25]. In both of them, CA was the most commonly isolated species throughout the years (64% in 2007 [10], and 56% in 2011 [25]). In another neighbouring country, Turkey, CA was found to make up 59.5% of the total strains in various departments at the Izmir Hospital [29]. Similarly in Italy, CA was the most commonly isolated species (72.7%) in different departments of a tertiary care hospital over a three-year period [2]. These results show that, although it is clear that the rate of recovery of NAC is increasing, CA remains the most common *Candida* species in general.

### Most commonly isolated NAC

The predominant speciated NAC in our study was *Candida glabrata* (59% of NAC, and 14% of total *Candida*). This finding was different from other Lebanese studies, in which *Candida tropicalis* was the most commonly isolated NAC (35–45% [10], and 20% [25], both of total isolates). This difference in species distribution between healthcare centres of the same country may be attributed to the selection bias used. In our study, we have included all non-faecal isolates of all departments, irrespective of their clinical significance, while in the two other studies [10, 25], the analysed isolates were retrieved from clinically relevant specimens, either when speciation was performed based on the treating clinicians’ requests [10], or in patients who warranted the use of antifungal therapy in special clinical circumstances [25].

Similar to our findings, *Candida glabrata* predominated among NAC in many centres, such as in France (15% of total) [8] and in Turkey (14.4% of total) [29]. Yet, other NAC may prevail in other settings. For example, *Candida krusei* was the most common NAC (12.9% of total *Candida*) in the Italian study, in which the majority of specimens were collected from patients with neutropenia [2]. Another example is the prevalence of *Candida tropicalis* in a tertiary care centre in India (46% of total) [30]. These incongruous findings of NAC distribution among different geographical zones, and even in different facilities in the same zone as in our country, highlight the importance of sample choice, antimicrobial prescription habits, the studied population, the unit(s) involved and the time frame of the study.

### Distribution of different Candida species among the departments

The difference in the isolation of different *Candida* species among the departments was statistically significant in our hospital (*P* = 0.005) (Table 5). CA was the most common among all departments, with the highest proportion in the OBGYN department (91.4%), followed by the paediatric department (84.8%). In the Italian study, CA was found to be the most common *Candida* species (72.7%), with the following distribution among the different departments: 64.2% in haematopoietic stem cell transplant units, 71.1% in the ICU, and 83.7% in paediatrics (*P* = 0.005 and 0.01 when compared to paediatrics respectively) [2].

With regard to NAC in our series, *Candida glabrata* prevailed in the IM department (15.7% of total *Candida*). Likewise, in the Turkish study, investigators found *Candida glabrata* as the most common NAC in all departments, mostly in the infectious diseases department (40% of NAC) [29].

In critical care, the most common speciated NAC after *Candida glabrata* in our hospital was *Candida famata* (12.4% of NAC), while in the study by Ece et al. [29], the most common NAC in the critical care/anaesthesiology department was *Candida krusei* (40% of NAC).

*Candida parapsilosis* is known to be a bloodstream isolate, and a common NAC in the paediatric population [31]. Our paediatric department had the highest rate of *Candida parapsilosis* (25% of NAC) compared to the other departments, but this was still less common than *Candida glabrata* (33% of NAC).

Therefore, the relative distribution of *Candida* species among different departments of the same hospital differs, with less CA in departments of the severely sick or immunocompromised patients. Among NAC, departments with critically ill and neutropenic patients had more non-*glabrata* NAC in their fungal ecology.

### Correlations between antifungal consumption and Candida species distribution

The correlation between antifungal consumption and *Candida* species distribution in the literature is scarce. In a prospective multicentre French surveillance program on yeast bloodstream infections implemented in the ICU, haematology and surgery departments, involving all age groups, Lortholary et al. found that the use of echinocandins decreased CA emergence from 56 to 21% relatively to NAC [32].

Our study revealed that increasing overall consumption of antifungals and specifically azoles correlated positively with NAC, especially *Candida glabrata.* The use of azoles may exert a selection pressure, suppressing CA and fostering the growth of NAC since they are active against CA more than NAC [32].

Dagi et al. [33] determined the minimum inhibitory concentration (MIC_90_) of different antifungals against 200 *Candida* spp. isolates from bloodstream infections between 2010 and 2013 at Selcuk University Hospital in Turkey. The MIC_90_ of antifungals against *Candida glabrata* was as such: 4 μg/mL for fluconazole, 0.12 μg/mL caspofungin and 0.06 μg/mL for anidulafungin. Based on this susceptibility of *Candida glabrata* to echinocandins, one would expect a negative correlation between them. However, our data did not show a negative correlation between the echinocandins and *Candida glabrata*. This lack of negative correlation may have been due to sampling bias whereby the majority of our specimens were from urine (Fig. 1). Despite moderate distribution of echinocandins into the kidneys, they exhibit negligible concentrations (< 2%) of intact drug in human urine. Thus, *Candida* growing in urine might not be affected by the use of echinocandins [34].

Unfortunately, antifungal susceptibility data of different *Candida* species is lacking in our study. This could have aided in interpreting Spearman’s correlations, especially that reports about echinocandin resistance in *Candida glabrata* have started to appear [35]. Alexander et al. tested the echinocandin susceptibility of all *Candida* species causing bloodstream infections between 2001 and 2010 at Duke University Hospital in the US and found that echinocandin resistance increased from 4.9 to 12.3% [35].

The Spearman’s coefficient showed a weaker positive correlation of amphotericin B with the emergence of NAC compared to azoles (SC = 0.27 and 0.38, respectively) and a negative correlation between amphotericin B and *Candida glabrata* (SC = − 0.003). This could be explained by the fact that amphotericin B, unlike azoles, is equally active against most *Candida* species, including CA and NAC (except *Candida dubliniensis*) or more specifically *Candida glabrata* [36]. So its use in wards, such as paediatrics and oncology, might have buffered the selection pressure exerted by the azoles in promoting the growth of NAC or *Candida glabrata*.

The isolation of non-speciated *Candida* and *Candida famata* in our series (2% of total isolates, and 9% of NAC) is noteworthy. The emergence of non-classical *Candida* is increasingly reported in the literature. Between 2012 and 2015, a new species called *Candida auris* was reported from 3 continents, South East Asia (Pakistan, India), South Africa and South America (Venezuela), and has shown resistance to several classes of antifungals [37]. This change in *Candida* species epidemiology is in no doubt driven by antifungal consumption and thus highlights the importance of implementing antifungal stewardship.

## Limitations and strengths

One limitation is that this study was single-centred, so our results could not be representative of the whole country. Another was the lack of antifungal susceptibility data in our facility that would have clarified the correlation between antifungal consumption and *Candida* species distribution. Another issue was that in some wards like the IM ward, some patients might have been clinically unstable necessitating ICU admission, or they might have been transferred early from the critical care unit when still unstable and on broad-spectrum antimicrobials due to shortage in ICU beds. These patients were counted as IM patients not as ICU patients. Consequently, the isolation of specific *Candida* species and antifungal consumption in the IM ward were affected by this occasional mixing of clinically stable and unstable patients. Nevertheless, this study is among the first studies in the region that describes antifungal consumption and relates it to *Candida* species distribution on hospital setting. We observed a clear difference in both elements among different wards, yet this difference would have been more significant if mixing in patient populations did not occur. In addition, this study describes overall *Candida* ecology in one facility rather than being limited to *Candida*-related bloodstream infections.

## Conclusion

Different *Candida* species are distributed unequally among the hospital departments of our facility, and this correlates with antifungal consumption. Our study highlights the need for benchmarking antifungal use, and standardisation of the metrics. Yet, the relationship between the changing *Candida* ecology according to the antifungal use highlights the strong need for antifungal stewardship to prevent reaching the era of predominant multi-drug resistant *Candida*.

## Abbreviations

ATC: Anatomical Therapeutic Chemical
CA: *Candida albicans*
DDD: Defined Daily Dose
DOT: Days of Therapy
ICU: Intensive Care Unit
IM: Internal Medicine
MGH: Makassed General Hospital
MIC: Minimum Inhibitory Concentration
NAC: *Non-albicans Candida*
OBGYN: Obstetrics/Gynaecology
PD: Patient Days
RDD: Recommended Daily Dose
SC: Spearman’s Coefficient
WHO: World Health Organisation
